# 

**Published:** 1895-05

**Authors:** 


### CONTENTS FOR MAY, 1895.

ORIGINAL COMMUNICATIONS. PAGE.

Nasal Stenosis. By S. MITCHELL, M. D. .... 577
Dangers of the Uterine Tampon. By WILLIS E. FORD, M. D. .... 588
Hospital Gangrene of Diphtheritic Origin—The Relation of Tubal Pregnancy to Pelvic Hematocele—Peritoneal Adhesions. By EMORY LANPHEAR, M. D. .... 592

PROGRESS IN MEDICAL SCIENCE.

State Medicine, Public Health, Hygiene and Bacteriology .... 596
Pediatrics .... 604

ABSTRACTS.

Complicated Hernia .... 607
A New Microörganism Discovered in Pork .... 608

STATE MEDICAL EXAMINATIONS.

Indorsement of Licenses from Other State Examining Boards .... 609

SELECTIONS.

To Avoid and to Prevent Consumption .... 611
Infected Feather Beds .... 613
The Strychnine Treatment of Pulmonary Consumption .... 614

EDITORIAL.

Vaginal Douching During the Puerperium .... 615
Topics of the Month .... 617

Personal .... 621
Hospital Notes .... 622
Society Meetings .... 622
Medical College Notes .... 626
Book Reviews .... 627
Literary .... 638
Miscellany .... 640

